# Characterising the psychopathological profile of children at high-risk for eating disorders

**DOI:** 10.1007/s00787-026-03005-7

**Published:** 2026-04-08

**Authors:** Stefana Aicoboaie, Bianca Borsarini, Edoardo Pappaianni, Stefania Konstantopoulou, Nadia Micali

**Affiliations:** 1 Center for Eating and feeding Disorders Research, Mental Health Center Ballerup, Copenhagen University Hospital – Herlev and Gentofte, Copenhagen, Denmark; 2 Institute of Biological Psychiatry, Mental Health Services, Amager and Hvidovre Hospital, Copenhagen University Hospital, Copenhagen, Denmark; 3 Research group “Spatial attention, perception and action”, Faculty of Medicine, Geneva University Neurocenter, University of Geneva, Geneva, Switzerland; 4 Division of Neurorehabilitation, Department of Clinical Neurosciences, Geneva University Hospitals, Geneva, Switzerland; 5 Great Ormond Street Institute of Child Health, University College London, London, UK

**Keywords:** Familial High Risk, Eating Disorders, Psychopathology, Intergenerational, Anorexia nervosa, Bulimia nervosa, Binge eating disorder, Offspring, Emotional disorders

## Abstract

Children at familial high-risk (FHR) for eating disorders (EDs), i.e. born to mothers with EDs, are at increased risk of psychiatric morbidity, especially emotional and behavioural disorders. However, less is known about broader psychopathology and specific effects of maternal diagnoses. This study aimed to characterise the psychopathological profile of children at FHR for EDs, compared to healthy children at no familial risk (HC, i.e., with a mother with no ED). Sixty (60) children at FHR (median age 10; range = 7) and 63 HC (median age 12; range = 4) were assessed for psychopathology using the Development and Well-Being Assessment, the State and Trait Anxiety Inventory, the Strength and Difficulties Questionnaire and the Social Aptitude Scale respectively. Children at FHR exhibited more psychiatric disorders than HC (*p*=.02 and *p*=.01 respectively*)*. Children at FHR showed more emotional disorders (*p*=.01) and overall more mental health difficulties, with elevated levels of emotional symptoms (*p*<.001), hyperactivity/inattention (*p*=.04), and peer relationship problems *(p*=.004). However, no significant differences were observed between groups in terms of state/trait anxiety. Moreover, post-pubertal development was associated with higher emotional distress and higher state anxiety levels than pre-pubertal development. Children at FHR are at increased risk of developing psychiatric disorders and psychopathology, especially in the emotional domain. Puberty may also be a critical window of risk for this group. Our findings suggest that children at FHR face an increased psychopathological burden and warrant development of interventions to support them, especially during puberty.

**Supplementary Information:**

The online version contains supplementary material available at https://doi.org/10.1007/s00787-026-03005-7.

## Introduction

Eating disorders (EDs) such as anorexia nervosa (AN), binge eating disorder (BED) and bulimia nervosa (BN) are debilitating psychiatric conditions with a strong psychological, physical, and social impact [1–3]. EDs aggregate in families, with first-degree relatives of ED patients having a three-to five-fold increased risk of an ED diagnosis themselves [4]. The risk is even higher (up to 11 times) in first-degree female relatives of AN individuals than in individuals without an AN relative [5]. Relatives of BN individuals are also at increased risk of developing BN themselves compared to individuals without a family relative with BN (between 4.4 [5]. to 9.6 [6] times greater risk) and BED is also thought to aggregate within families [7], independent of obesity [8].

The observed familial aggregation is explained by both genetic (i.e., parents passing on genes that increase risk for EDs) and environmental (e.g., parents modelling an environment that fosters the development of an ED) factors that interplay to increase risk of ED in offspring of mothers with a history of an ED. Recent heritability estimates underscore the genetic contribution to ED: at the population level, genetics account for about 36% of the variation in AN expression and 39% of the variation in BED expression [4]. Additionally, children can learn eating behaviours and attitudes from their mothers. One study showed that children as young as 10 old already show dietary restraints when their mothers have a similar ED history [9]. Thus, the interplay of inherited susceptibility and learned eating habits can together perpetuate the intergenerational transmission of EDs.

Although much of the attention has been on the development of ED in offspring of mothers with a history of an ED, familial risk is not restricted to homotypic continuity (i.e., offspring developing the same parental diagnosis) as children are also at an increased risk for other psychiatric conditions (i.e., heterotypic transmission) [10]. A recent systematic review concluded that children of mothers with an ED are more likely to experience negative developmental outcomes such as difficult temperament and emotional, behavioural, and cognitive disturbances than healthy controls (HC) [11], i.e. children of mothers with no psychiatric disorders. Mothers report difficult infant temperament (e.g., child cries a lot, demands a lot of attention) as early as 6 months postpartum, yet it is not clear whether the infants of mothers with an ED are objectively more difficult or whether mothers are likely to read their offspring’ behaviour as emotionally negative [12]. Moreover, because temperament has biological underpinnings and is genetically influenced [13], a difficult temperament in children may reflect an inherited predisposition. Disorder-specific, community-based research suggests that children of mothers with AN are more likely to present emotional difficulties, while children of mothers with BN are more likely to exhibit hyperactivity and conduct and peer problems [14].

While previous studies mainly focused on the effects of maternal AN or BN on child psychopathology in population-based cohorts (see e.g. [15]), the present clinical-population study focused exclusively on mothers with a lifetime ED diagnosis and the well-being of their offspring. Compared to previous work that employed a broad recruitment strategy and were based on women who were not treatment-seeking and not diagnosed using clinical interviews (e.g., the Avon Longitudinal Study of Parents and Children recruited all pregnant women living in the Avon region, United Kingdom, who had their due date between April 1 st 1991 and December 31 st 1992, answered, as part of a broader mental health assessment, questions on lifetime history of AN and BN [15]), the current study focused solely on mothers with a clinician-assessed ED diagnosis (AN, BN or BED) and who were for the majority treatment seeking. This approach guarantees the specificity of the psychiatric diagnoses and allows for a comprehensive clinical assessment.

We set to characterise the psychopathological profile of children at familial high risk (FHR) (i.e., born to mothers with EDs) for ascertained AN, BED or BN assessing both diagnoses and symptomatology during a developmental period of heightened psychiatric risk. Based on the available literature, we hypothesised that children at FHR for EDs would present more psychiatric morbidities and have more emotional and behavioural difficulties than HC. Given the high rates of comorbidity between ED and anxiety disorders [16], we were interested in whether children at FHR for ED would also be susceptible to experiencing anxiety. We also explored the effect of maternal ED diagnosis on outcomes, and prediction of child disorder based on maternal psychopathology and child characteristics.

## Methods

### Design and participants

The Neurobiology and Cognition in Eating Disorders: A High-Risk Eating Disorder Offspring Study (eNCORE) is a cross-sectional study investigating trait markers of ED in young (8 to 15 years old) offspring at FHR for ED (for a complete description of the study see [17]). Mothers with a lifetime history of an ED and their healthy offspring were recruited from ED treatment centers and a private clinic in Geneva and the adjacent Vaud canton between November 2019 and July 2022; control mothers and children were recruited from the same geographic area during the same period via flyers and social‑media advertisements. Mothers had to meet diagnostic criteria for lifetime diagnosis (past or current) of an ED (AN, BN or BED) according to the 5th edition of the Diagnostic and Statistical Manual of Mental Disorders (DSM-5) [2]; or to be included in the HC group, they had to have no history of psychiatric conditions, including ED. Offspring were excluded from participating in the study if they reported ED symptoms with impact or subthreshold/full-blown ED, a history of neurological disorders, and intake of medication affecting the central nervous system [17]. We decided to exclude offspring who reported any ED symptoms as custom in high-risk studies, given the aim of characterising the psychopathological profile of children at risk for EDs *prior to any ED onset*, so as to identify traits that are associated with being at risk for an ED but are not confounded by the presence of an ED. We excluded two children from the FHR group and one child from the HC due to presence of ED symptoms, such as binge eating, with impact. The final sample consisted of 60 FHR (46 girls, 76.67%) and 63 HC (50 girls, 79.36%) children.

### Ethics

Ethical approval was received by the Swiss Association of Research Ethics Committees (https://swissethics.ch/en (ID 2018–02363)). Both mothers and children older than 14 provided informed consent for participation. The younger children verbally consented to participate in the study after being introduced to the experimental procedures.

## Measures

### Socio demographic data

Maternal age, ethnicity, educational attainment, as well as offspring age, were collected via ad-hoc questionnaires, weight and height (to calculate body-mass index (BMI) and weight for height), and pubertal status (assessed using pictograms to determine Tanner Stages [18]) were collected with objective measurements for children.

Mother psychopathology was assessed with the Structured Clinical Interview for DSM‑5 Research Version (SCID‑5‑RV) general checklist and its Eating Disorders module [19–21], both administered in French by a trained research assistant/PhD student (BB).

### Offspring psychopathology

Presence of psychiatric disorders was evaluated with the Development and Well-Being Assessment (DAWBA) [22]. The DAWBA is a semi-structured interview validated for psychiatric disorders [22] (according to the 10th version of the International Statistical Classification of Diseases and Related Health Problems (ICD-10) [23] and the 4th or 5th edition of DSM [24]). The DAWBA was completed by mothers for children below the age of 11 (*N* = 47 – 16 controls and 31 cases) and by mothers and children for those above 11 years of age (*N* = 76 – 47 controls and 29 cases). As usually done in clinical settings, diagnoses derived from multi-informant DAWBA computerized algorithms and transcripts were reviewed by a trained rater (NM), blind to child status (FHR or HC) who confirmed or overturned diagnoses. Our outcomes of interest were: any ICD-10 Diagnosis, any DSM-5 diagnosis, presence of any (either ICD-10 or DSM-5) emotional (i.e., emotional, separation anxiety, specific phobia, social phobia, panic disorder, agoraphobia, post-traumatic stress disorder, obsessive-compulsive disorder, generalized anxiety, other anxiety, depressive episode, major depression, other depression, undifferentiated anxiety/depression, mania/bipolar) or behavioural disorder (i.e., Attention deficit hyperactivity disorder (ADHD), ADHD combined, ADHD inattentive, ADHD hyperactive-impulsive, hyperactivity, other hyperactivity, hyperkinesis, conduct/oppositional, oppositional defiant, conduct disorder, other disruptive). Psychosis, tic disorder, stereotypic disorder, PDD/autism diagnoses were categorised as: “Other disorders”.

Dimensional psychopathology was assessed using the Strength and Difficulties Questionnaire (SDQ [25, 26]). The SDQ is a widely used, well-validated screening instrument used in the assessment of childhood psychopathology. It consists of five scales, each consisting of five items, that assess emotional problems, conduct problems, hyperactivity, peer problems, and prosocial behaviour, and an “impact supplement”. Additionally, a total difficulties score is computed by summing the scores of all scales except the prosocial scale. The impact score is generated by summing the distress and impairment items.

ASD traits were measured by the Social Aptitude parent report Scale (SAS) that is part of DAWBA [22]. The SAS consists of 10 items that check for social understanding and social abilities (e.g., aptitudes that reflect a good and rapid understanding of social and environmental cues, in a complex situation, that inform socially appropriate behaviours). Higher SAS scores reflect better social aptitudes [27].

Self-reported state and trait anxiety levels were assessed using the validated State and Trait Anxiety Inventory (STAI) [28] completed by children/adolescents. The inventory consists of 20 questions assessing state anxiety (i.e., anxiety experienced in the present moment) and 20 questions assessing trait anxiety (i.e., anxiety experienced long term).

## Statistical analysis

### Offspring psychopathology

Our primary analyses consisted of between group comparisons (FHR vs. HC) in terms of child psychopathology (diagnoses, dimensional psychopathology, ASD and STAI) and logistic regressions looking at whether maternal diagnosis, age and education, and child pubertal development associate with child psychopathology (diagnoses and STAI).

We additionally ran some post-hoc exploratory analyses stratifying the group comparisons by maternal diagnosis and some exploratory logistic regressions looking at the association between maternal internalising disorders (i.e., anxiety, depression, obsessive-compulsive disorder) and offspring emotional problems and between maternal externalising disorder (i.e., ADHD) and any behavioural problems in offspring.

#### Between group comparisons

The distribution of all variables was inspected for inconsistencies and outliers. Demographic variables and psychopathology were compared between groups using either Chi-Square Test or Fisher’s exact test for categorical variables and Independent Samples t-Test or Mann-Whitney U test for continuous variables. All significant analyses were further explored by running post-hoc analyses stratified by maternal diagnosis. We ran additional ordinal logistic regression (using the *MASS* package in R [29]) to account for the age difference between the groups in terms of dimensional psychopathology (i.e., SDQ scores).

We reported effect sizes (Phi Φ for 2 × 2 contingency tables or Cramer’s V for larger contingency tables and Cliff’s Delta (δ) for non-parametric t-tests with unequal sample size) for all performed analyses.

Given that women are more commonly affected by an ED than men during their lifetime (women: between 2.6 and 8.4% of the global population; men: between 0.7 and 2.2% of the global population [30–32], we ran our analyses both on the combined (i.e., female and male offspring) and female-offspring only sample.

#### Logistic regressions

Binary logistic regressions were run in the female only sample to assess whether maternal diagnosis, age and education, and child pubertal development accounted for offspring psychopathology (DAWBA and STAI).

STAI scores were dichotomised into high-STAI (i.e., scores in the top quartile of the distribution) or low-STAI (i.e., scores not in the top quartile of the distribution) scores. Given the low cell count for “Any Behavioural”, we performed a lasso-penalized logistic regression with 10-fold cross-validation for regularisation parameter tuning in RStudio using the *glmnet* package [33].

Given the exploratory nature of our research question, we report both unadjusted and Bonferroni-adjusted analyses. The analyses were corrected for multiple comparisons by dividing the alpha level (α = 0.05) by the number of pairwise comparisons per outcome.

All analyses were performed in SPSS version 29.0.1.0 [34] and in RStudio 2024.04.02 [35].

## Results

### Socio-demographic characteristics

The two groups were statistically different in terms of age (U = 1202.5, *p* <.001), with HC children being on average older than children at FHR, and weight for height (U = 2353, *p* =.02), with HC children having a higher weight for height than children at FHR. Sex distribution was comparable between the two groups (*p* =.83). Additionally, HC mothers were significantly older than mothers with a lifetime history of EDs (U = 1151.5, *p* <.001) (Table 1).

**Table 1:** Sociodemographic characteristics

	FHR (*N* = 60)	HC (*N* = 63)	Statistics
Child age(Median, Range)	10, 7	12, 4	U=1202.5p <.001*
Biological sex (females)	46 (76.67%)	50 (79.36%)	*FET = 0.83*
Maternal Diagnosis			
*Anorexia Nervosa*	27 (45%)		
*Binge Eating Disorder*	20 (33.33%)		
*Bulimia Nervosa*	13 (21.67%)		
Ethnicity (White European)	53 (88.33%)	51 (80.95%)	*X*^*2*^(4, *N* = 123) = 4.75, *p* =.31
Weight for Height (Median, Range)	97.83, 80	105.15, 80.92	U = 2353 *p* =.02*
Female Tanner stage (breast development) Pre-pubertal stage Post-pubertal stage	35 (76.09%) 11 (23.91%)	28 (58.33%) 20 (41.67%)	*X*^*2*^(1, *N* = 94) = 3.35, *p* =.08
Maternal age (years) (Median, Range)	42, 21	45, 28	U = 1151.5, *p* <.001*
Maternal Education Up to and including secondary school University and higher	24 36	19 44	*X*^*2*^(1, *N* = 123) = 1.31, *p* =.26

FHR: Familial High-Risk HC: Healthy Controls; *: p < = 0.05

### Offspring psychopathology

#### Diagnoses

There were statistically significant associations of small to medium effect between FHR status and “Any ICD-10” or “Any DSM-5” disorder (*X*^2^ = 5.47, *p* =.02, Phi (Φ) = 0.23 and *X*^2^ = 6.12, *p* =.01, Phi (Φ) = 0.24 respectively) and “Any Emotional Disorder” (*X*^2^ = 6.38, *p* =.01, Phi (Φ) = 0.25) (Table 2).

**Table 2:** Psychiatric disorders in children at familial risk for eating disorders and healthy controls as assessed by the DAWBA: p values and effect sizes from Chi-Square Test or Fisher’s exact tests

	Whole sample				Female sample			
	FHR (*N* = 59)	HC (*N* = 62)	Statistics	Effect size	FHR (*N* = 46)	HC (*N* = 49)	Statistics	Effect size
Any ICD-10 Disorder	20 (33.89%)	9 (14.52%)	*X*^2^ (1, *N* = 121) = 5.47, *p* =.02*	Phi (Φ) = 0.23	18 (39.13%)	6 (12.25%)	*X*^2^ (1, *N* = 94) = 8.10, *p* =.004*	Phi (Φ) = 0.32
Any DSM-5 Disorder	21 (35.59%)	9 (15.51%)	*X*^2^ (1, *N* = 121) = 6.12, *p* =.01*	Phi (Φ) = 0.24	19 (41.30%)	6 (12.25%)	*X*^2^ (1, *N* = 95) = 8.89, *p* =.002*	Phi (Φ) = 0.33
Any Emotional Disorder	16 (27.12%)	5 (8.06%)	*X*^2^ (1, *N* = 121) = 6.38, *p* =.01*	Phi (Φ) = 0.25	15 (32.61%)	5 (10.20%)	*X*^2^ (1, *N* = 95) = 5.88., *p* =.01*	Phi (Φ) = 0.28
Any Behavioural Disorder	6 (10.17%)	3 (4.84%)	*FET* = 0.31	Phi (Φ) = 0.10	5 (11.11%)	0	*FET* = 0.02*	Phi (Φ) = 0.24
Other Disorder	4 (6.78%)	1 (1.61%)	*FET = 0.20*	Phi (Φ) = 0.13	3 (6.52%)	1 (2.04%)	*FET* = 0.35	Phi (Φ) = 0.11

*FHR: Familial High-Risk for eating disorders;*

*HC: Healthy Controls*;

*: p < = 0.05

When stratifying the analysis by ED subtype, there were associations between maternal history of BED and BN and “Any Emotional Disorder” (*p* =.03 and *p* =.01 respectively), but these associations did not remain significant after adjusting for multiple comparisons (Supplementary Table 1).

##### Females only

There were statistically significant associations of medium effect size between FHR status and “Any ICD-10” or “Any DSM-5” disorder (*X*^2^ = 8.10, *p* =.004, Phi (Φ) = 32 and *X*^2^ = 8.89, *p* =.002, Phi (Φ) = 0.33 respectively), “Any Emotional Disorder” (*X*^2^ = 5.88, *p* =.01, Phi (Φ) = 0.28) and “Any Behavioural Disorder” (*FET* = 0.02, Phi (Φ) = 0.24) with females at FHR for ED showing higher psychopathology than HC (Table 2).

When stratifying the analysis by ED subtype there was, after adjusting for multiple comparisons, a statistically significant association of large effect size between maternal history of AN and “Any Behavioural Disorder” (FET = 0.003, Phi (Φ) = 0.45) (Supplementary Table 1).

#### Dimensional psychopathology

There was a statistically significant difference of large effect size between risk status and emotional symptoms (U = 2630.5, *p* <.001, Cliff’s Delta (δ) = − 0.49) and several significant associations of small to moderate effect size between risk status and hyperactivity, peer problems (U = 2157, *p* =.04, Cliff’s Delta (δ) = − 0.22 and U = 2290.5, *p* =.004, Cliff’s Delta (δ) = − 0.29 respectively), total difficulties (U = 2518, *p* <.001, Cliff’s Delta (δ) = − 0.42) and impact scores (U = 2362.5, *p* <.001, Cliff’s Delta (δ) = − 0.33) with children at FHR for ED showing on average higher levels of psychopathology than HC (Table 3). The age difference between the groups did not explain the observed difference between the groups (Supplementary Table 4).

**Table 3:** Mental health symptoms shown as median scores on the SDQ in children at familial risk for eating disorders and healthy controls: p values and effect sizes from Mann-Whitney U test

	Whole sample				Female sample			
	FHR (*N* = 60)	HC (*N* = 59)	Statistics	Effect size	FHR (*N* = 46)	HC (*N* = 47)	Statistics	Effect size
Emotional symptoms score, Median (Range, Min-Max)	4 (10, 0–10)	1 (8, 0–8)	*U* = 2630.5, *p* <.001*	δ = − 0.49	4 (10, 0–10)	1 (8, 0–8)	*U* = 1653.5, *p* <.001*	δ = − 0.53
Conduct problems score, Median (Range, Min-Max)	1 (7, 0–7)	1 (5, 0–5)	*U* = 1940, *p* =.35	δ = − 0.10	1 (6, 0–6)	1 (4, 0–4)	*U* = 1096.5, *p* =.90	δ = − 0.01
Hyperactivity score, Median (Range, Min-Max)	3 (10, 0–10)	2 (10, 0–10)	*U* = 2157, *p* =.04*	δ = − 0.22	2.5 (10, 0–10)	2 (6, 0–6)	*U* = 1293.5, *p* =.10	δ = − 0.20
Peer problem score, Median (Range, Min-Max)	1 (5, 0–5)	0 (5, 0–5)	*U* = 2290.5, *p* =.004*	δ = − 0.29	1 (5, 0–5)	0 (5, 0–5)	*U* = 1326, *p* =.05*	δ = − 0.23
Pro social score, Median (Range, Min-Max)	9 (7, 3–10)	9 (6, 4–10)	*U* = 1966, *p* =.28	δ = − 0.11	9 (7, 3–10)	9 (6, 4–10)	*U* = 1243.5, *p* =.19	δ = − 0.15
Impact score, Median (Range, Min-Max)	0 (9, 0–9)	0 (6, 0–6)	*U* = 2362.5, *p* <.001*	δ = − 0.33	0 (9, 0–9)	0 (4, 0–4)	*U* = 1421, *p* < 001*	δ = − 0.31
Total difficulties score, Median (Range, Min-Max)	10 (25,0–25)	6 (21, 0–21)	*U* = 2518, *p* <.001*	δ = − 0.42	9.5 (24, 0–24)	6 (15, 0–15)	*U* = 1529 *p* <.001*	δ = − 0.41

*FHR: Familial High-Risk for eating disorders;*

*HC: Healthy Controls*;

*: p < = 0.05;

δ : Cliff’s Delta

When stratifying the analysis by ED subtype and adjusting for multiple comparisons, there were statistically significant differences between children of mothers with a lifetime history of AN, BED or BN and HC in terms of emotional symptoms (U = 479.5, *p* =.02, Cliff’s Delta (δ) = − 0.40; U = 307.5, *p* <.01, Cliff’s Delta (δ) = − 0.48 and U = 122.5, p = < 0.01, Cliff’s Delta (δ) = − 0.68 respectively), with children at FHR for ED showing higher levels than HC. Additionally, there was a statistically significant difference of moderate to large effect sizes between children of mothers with a lifetime history of AN and HC in terms of hyperactivity (U = 434, *p* <.01, Cliff’s Delta (δ) = − 0.45). Children of mothers with a lifetime history of BED were also statistically significant different than HC in terms of peer problems (U = 339.5, *p* =.02, Cliff’s Delta (δ) = − 0.42). Overall, children of mothers with a lifetime of AN or BED showed significantly higher total difficulties score than HC (U = 427, *p* <.01, Cliff’s Delta (δ) = − 0.46) (Supplementary Fig. 2). In terms of impact score, there was a statistically significant difference between children of mothers with a lifetime history of AN and HC (U = 482.5, *p* <.01, Cliff’s Delta (δ) = − 0.39).

##### Females only

There was a statistically significant difference of large effect size between risk status and emotional symptoms (U = 1653, *p* <.001, Cliff’s Delta (δ) = −53) and several significant associations of small to moderate effect size between risk status and peer problems (U = 1326, *p* =.05, Cliff’s Delta (δ) = − 0.23), total difficulties (U = 1529, *p* <.001, Cliff’s Delta (δ) = − 0.41) and impact scores (U = 1421, *p* <.001, Cliff’s Delta (δ) = − 0.31) (Table 3). The age difference between the groups did not explain the observed difference between the groups (Supplementary Table 4).

When stratifying the analysis by ED subtype and adjusting for multiple comparisons, we observed similar patterns to the whole sample analysis apart from the association between children at FHR for BED and peer problems (Supplementary Table 3).

#### ASD symptoms

There were no significant differences between children at FHR for ED and HC in terms of ASD symptoms (*p* =.59). No significant results were observed in the female only group.

#### State and trait anxiety

There were no significant differences between children at FHR for ED and HC in self-reported state (U = 1139, *p*=.94) and trait anxiety levels (U = 1179, *p*=.83) (Table 4). There were no significant differences between girls at FHR for ED and HC in self-reported state (U = 621, *p* =.93) and trait anxiety levels (U = 681.5, *p* =.55) (Table 4).

**Table 4:** State and trait anxiety in children at familial risk for eating disorders and healthy controls: p values and effect sizes from Mann-Whitney U test

	Whole sample				Female sample			
	FHR (*N* = 58)	HC (*N* = 57)	Statistics	Effect size	FHR (*N* = 45)	HC (*N* = 44)	Statistics	Effect size
STAI state, Median (Range, Min-Max) STAI trait, Median (Range, Min-Max)	31 (17, 22–39) 31 (46, 21–67)	30 (24, 22–46) 32.50 (28, 22–50)	*U* = 1139, *p* =.94 *U* = 1179, *p *=.83	δ = − 0.01 δ = 0.03	31 (16, 23–39) 29 (46, 21–67)	30 (24, 22–46) 32 (28, 22–50)	*U* = 621, *p* =.93 *U* = 681.5, *p* =.55	δ = − 0.01 δ = 0.08

*FHR: Familial High-Risk for eating disorders;*

*HC: Healthy Controls;*

δ : Cliff’s Delta

#### Maternal internalising disorder and emotional disorder in offspring

There was no significant association between maternal internalising disorder and any emotional problems in offspring (*p* =.75).

#### Maternal externalising disorder and behavioural disorder in offspring

There was no significant association between maternal externalising disorder and any behavioural problems in offspring (*p* =.11).

#### Predictors of psychopathology in FHR girls

Only maternal ED diagnosis (i.e., ED or HC) was significantly associated with presence of “Any ICD-10” and “Any DSM-5” Disorder (Odds Ratio [OR] = 6.96, [95% Confidence Interval (CI) = 2.11, 27.65], *p* =.003 and OR = 7.14[95%CI = 2.16–28.55], *p* =.002 respectively) suggesting that individuals at FHR are between 6.96 and 7.14 more likely to show any psychopathology compared to individuals at non-FHR (i.e., HC). No other predictors were identified (Table 5).

**Table 5:** Predictors of psychopathology in female only offspring (*N* = 96): Odds Ratios (ORs) and 95% Confidence intervals (CI) from logistic regression analyses

	Any ICD-10 Disorder		Any DSM-5 Disorder		Any Emotional Disorder		Any Other Disorder		STAI State		STAI Trait	
	OR [95%]	*p*	OR [95%]	*p*	OR [95%]	*p*	OR [95%]	*p*	OR [95%]	*p*	OR [95%]	*p*
*FHR*	6.96 [2.11–27.65]	0.003*	7.14 [2.16–28.55]	0.002*	6.26 [1.70–28.67]	0.01*	3.55 [0.31–88.68]	0.34	0.71 [0.19, 2.51]	0.60	1.15 [0.33, 4.02]	0.83
*Maternal age*	1.00 [0.89–1.11]	0.94	0.99 [0.88–1.09]	0.81	0.96 [0.86–1.08]	0.51	0.99 [0.81–1.23]	0.99	0.92 [0.80, 1.04]	0.18	1.04 [0.92, 1.18]	0.49
*Maternal education*	0.51 [0.16–1.62]	0.24	0.49 [0.16–1.55]	0.22	0.59 [0.18–2.07]	0.40	0.0.44 [0.04–4.93]	0.47	3.86 [0.88, 24.00]	0.10	1.01[0.27, 4.17]	0.99
*Pubertal development*	2.39 [0.70–8.70]	0.17	2.65 [0.81–9.45]	0.11	3.80 [1.09–14.61]	0.04*	2.36 [0.24–23.53]	0.44	5.00 [1.35, 21.01]	0.02*	3.60 [1.06, 12.91]	0.04

*FHR: Familial High Risk for eating disorders;*

*: p < = 0.05

##### Emotional disorders

Maternal ED diagnosis and pubertal stage were significantly associated with presence of any emotional disorder (OR = 6.26[95%CI = 1.70, 28.67], *p* =.01 and OR = 3.80[95%CI = 1.09, 14.61], *p* =.04). No other factors were significantly associated with presence of any emotional disorder (Table 5).

##### Behavioural disorders

Maternal ED diagnosis was associated with 6.81 higher odds of any behavioural disorder.

##### Other disorders

No statistically significant predictors were identified for the presence of any other disorder (Table 5).

##### State and trait anxiety

Pubertal stage was associated with both state and trait anxiety scores in the top quartile of the distribution (OR = 5.00[95%CI = 1.35, 21.11], *p* =.02; OR = 3.60[95%CI = 1.06, 12.91], *p* =.04 respectively), with individuals in the post-pubertal stage being 5 and respectively 3.60 times more likely to experience high state and trait anxiety levels compared to pre-pubertal. Neither groups status, maternal age, nor maternal education predicted high state anxiety levels (see Table 5).

## Discussion

Our clinical-population study focused on characterising, using gold standard assessment tools, both the mental health disorders and dimensional psychopathology in children at FHR for EDs, as well as explore the role of maternal ED and other diagnosis on child psychopathology. We identified notable differences in diagnoses and dimensional psychopathology between children at FHR for ED and HC that are in line with previous reports (see [11, 14]). In line with our hypotheses, children at FHR for EDs showed greater overall psychiatric morbidity, particularly emotional disorders, compared to HC. Our analyses suggest that the observed differences in FHR psychopathology are driven by psychopathology manifested in girls. Girls at FHR for EDs showed higher levels of behavioural disorders along overall greater psychopathology (specifically emotional disorders) compared to HC.

Children at FHR for ED also showed more mental health symptoms across domains (externalising and internalising) than HC. On average, their scores fell in the normal range of reports of hyperactivity, peer problems, as well as impact score and total difficulties scores, despite the association between risk status and the different SDQ subscales. This might suggest that while the difficulties experienced by children a FHR for ED are higher than HC, this might not always reach a level of clinical relevance. This prompts for careful observation and might index a prodromal phase in children at FHR for ED.

Our exploratory analyses of the role of maternal ED diagnosis on child psychopathology also provided interesting inferences. Contrary to expectations, daughters at FHR for AN were more likely to present with behavioural disorders and hyperactivity compared to HC. This could be explained by a combination of child psychopathological factors (e.g., fear, anxiety, impulsivity), environmental events (e.g., trauma) and mother-child relation [36]. Moreover, irritability is a transdiagnostic trait which should receive further scrutiny in future investigations as it could inform about risk for internalising disorders [37]. Whilst we identified higher levels of emotional disorders and related symptoms in children at FHR for EDs, self-reported state and trait anxiety did not differ across FHR and HC. This discrepancy raises interesting questions about inherent differences in the assessment tools vs. maternal/child perception of emotional problems. In fact, emotional disorders, as assessed by the DAWBA, include anxiety and depressive disorders, while the STAI focuses just on anxiety. In a recent study of a clinical sample, DAWBA anxiety disorder diagnosis strongly predicted child and parents reported anxiety [38]. The discrepancy might also stem from differences in informant: while the STAI is a self-report assessment, the DAWBA incorporates both parent and child report (for children above 11 years of age). While mothers with a lifetime history of mental illness might be more likely to overreport mental health problems in their offspring [39], adolescent might be better at reporting internalising symptoms than their parents [40].

Maternal history of internalising or externalising disorder was not associated with emotional or behavioural disorder in offspring, probably due to low power/low prevalence of disorders in children and mothers. However, maternal history of ED was associated with higher risk of psychopathology in female offspring, particularly emotional disorders. Interestingly, post-pubertal FHR children were at higher risk for emotional disorders and higher state and trait anxiety levels than pre-pubertal children. Therefore, puberty might be a particularly delicate time for the potential development of anxiety/emotional disorders in FHR girls. Our results suggest that puberty might be a critical window for girls at FHR for ED who might need targeted prevention during this transition period since puberty is also known to be a risk factor for the emergence of EDs and EDs symptoms particularly in girls [41].

### Strengths and limitations

While the current literature primarily focuses on population-based cohorts, the present study offers a comprehensive view on the psychopathological profile of children at FHR for ED by employing a range of assessments, including a semi-structured interview and validated multi-informant measures.

Several limitations need to be acknowledged. Although some of the associations passed the test of statistical significance, the wide confidence intervals warrant caution in interpreting the results. Our analysis suffered from a relatively small sample size and a low event rate; thus, the reported results call for additional exploration. The study provides a cross-sectional view on the mental health well-being of children at FHR for ED. Longitudinal studies are further needed to better understand the psychopathological trajectories of these children. Moreover, we were unable to conclusively study the psychopathological profile of males at FHR for ED as our sample mainly consisted of girls (approximately 80% of the sample). Thus, future studies should aim for more representative samples (e.g., include more males, diverse sexual and ethnic minorities) as these groups might face different risks [42, 43]. Moreover, as much as the project intended to recruit randomly sampled HC, we cannot exclude the possibility that ours are self-selected HC and FHR samples (e.g., participate because they are worried about their offspring wellbeing and risk status) which might have biased the levels of observed psychopathology. The prevalence of DAWBA diagnoses in HC, however, suggests the HC sample was representative of the general population (i.e., not ‘supercontrols’). Moreover, given the self-reported nature of our assessments, the assessments are biased by response biases [44]. This is of relevance particularly for our at-risk sample, in that mothers with psychiatric disorders might be more prone to report difficulties or worry about their children. This is an important consideration given that psychopathology was reported by mothers (due to instrument used) for FHR girls (31 vs. 16 in the HC group). Group differences may therefore be influenced by the type of reporter. Finally, future research should also account for paternal effects for a holistic understanding of the patterns of ED inheritance. Although most of the literature has focused on the effects of maternal ED on child’s well-being, evidence shows that fathers’ body dissatisfaction (a core component of EDs) correlates with monitoring their sons’ but not daughters’ food intake [45] suggesting that certain pattern of inheritance maybe be dyad specific (father-son, mother-daughter).

The mechanisms behind increased psychopathology in children at FHR still need further investigation, however there is evidence of shared genetic risk between EDs and other psychiatric disorders [4]. Our observation of higher levels of emotional and hyperactivity/inattention problems in children at FHR for ED compared to HC might be explained by the large genetic correlation between EDs and ADHD and anxiety disorders. Future research should take into consideration other risk factors too (e.g., gut microbiota, socio-economic status, social media, parenting style) that can build a more comprehensive and sensitive risk profile for EDs.

We are currently undergoing a large longitudinal multi-modal study (e.g., clinical, cognitive, genetic, neuroimaging) that aims to better understand the factors that confer risk as well as resilience to the development of ED [46].

## Conclusion

Children at FHR for ED exhibit significantly higher levels of psychopathology, especially in the emotional domain, and are at increased risk of developing psychiatric disorders. Additionally, our results postulate that puberty may be a critical window of risk. These findings underscore the need for early intervention strategies to mitigate the heightened emotional burden faced by these children with particular focus on supporting them during their pubertal development. Moreover, longitudinal studies are warranted to better characterise the developmental trajectories of children at FHR for EDs and potentially provide insight into early screening and prevention of EDs and associated morbidities.

## Supplementary Information

Below is the link to the electronic supplementary material.

Supplementary Material 1 (DOCX 514 KB)

## Data Availability

Available on reasonable request.
