# Supplementary material for: Characterising the psychopathological profile of children at high-risk for eating disorders
Source: Eur Child Adolesc Psychiatry. 2026 Apr 8;35(8):2477–86. doi: 10.1007/s00787-026-03005-7 (PMC13652771; doi:10.1007/s00787-026-03005-7)
Supplement: Supplementary file 1 [file 787_2026_3005_MOESM1_ESM.docx]

S1*. Effect of specific maternal ED diagnoses on child psychiatric outcomes: p values and effect sizes from Chi-Square Test or Fisher’s exact tests*

|  |  | *Whole sample* | | *Female sample* | |
| --- | --- | --- | --- | --- | --- |
| **Psychiatric disorders in offspring** | **Maternal diagnosis** | **Statistics** | **Effect size** | **Statistics** | **Effect size** |
| AnyICD-10 | AN vs no ED | *X*^2^ (1, *N* = 88) = 2.15, p = .14 | Phi (Φ) = .19 | *X*^2^ (1, *N* = 65) = 3.57, p = .06 | Phi (Φ) = .28 |
|  | BED vs no ED | *X*^2^ (1, *N* = 82) = 2.84, p = .09 | Phi (Φ) = .22 | *X*^2^ (1, *N* = 66) = 4.97, p = .03 | Phi (Φ) = .32 |
|  | BN vs no ED | *X*^2^ (1, *N* = 74) = 3.22, p = .07 | Phi (Φ) = .26 | *X*^2^ (1, *N* = 61) = 3.83, p = .05 | Phi (Φ) = .30 |
|  | AN vs BED | *X*^2^ (1, *N* = 47) = <.01, p = 1 | Phi (Φ) = .05 | *X*^2^ (1, *N* = 34) = <.01, p = 1 | Phi (Φ) = .04 |
|  | AN vs BN | *X*^2^ (1, *N* = 39) = .08, p = .77 | Phi (Φ) = .11 | *X*^2^ (1, *N* = 29) = < .01, p = 1 | Phi (Φ) = .04 |
|  | BED vs BN | *X*^2^ (1, *N* = 32) = <.01, p = 1 | Phi (Φ) = .07 | *X*^2^ (1, *N* = 29) = <.01, p = 1 | Phi (Φ) = .005 |
| Any DSM-5 | AN vs no ED | *X*^2^ (1, *N* = 88) = 3.04, p = .08 | Phi (Φ) = .22 | *X*^2^ (1, *N*=65) = 4.97, p = .03 | Phi (Φ) = .32 |
|  | BED vs no ED | *X*^2^ (1, *N* = 82) = 2.84, p = .09 | Phi (Φ) = .22 | *X*^2^ (1, *N* = 66) = 4.97, p = .03 | Phi (Φ) = .32 |
|  | BN vs no ED | *X*^2^ (1, *N* = 74) = 3.22, p = .07 | Phi (Φ) = .26 | *X*^2^ (1, *N* = 61) = 3.83, p = .05 | Phi (Φ) = .30 |
|  | AN vs BED | *X*^2^ (1, *N* = 47) = <.01, p = 1 | Phi (Φ) = .02 | *-* | - |
|  | AN vs BN | *X*^2^ (1, *N* = 39) = .02, p = .89 | Phi (Φ) = .08 | *X*^2^ (1, *N* = 29) = <.01, p = 1 | .005 |
|  | BED vs BN | *X*^2^ (1, *N* = 32) = <.01, p = 1 | Phi (Φ) = .07 | *X*^2^ (1, *N* = 29) = <.01, p = 1 | .005 |
| Any Emotional Disorder | AN vs no ED | *X*^2^ (1, *N* = 89) = 1.14, p = .28 | Phi (Φ) = .15 | *FET* = .22 | Phi (Φ) = .17 |
|  | BED vs no ED | *X*^2^ (1, *N* = 82) = 4.52, p = .03 | Phi (Φ) = .27 | *X*^2^ (1, *N* = 66) = 4.06, p = .04 | Phi (Φ) = .29 |
|  | BN vs no ED | *X*^2^ (1, *N* = 74) = 7.05, p = .01 | Phi (Φ) = .36 | *X*^2^ (1, *N* = 61) = 4.86, p = .03 | Phi (Φ) = .34 |
|  | AN vs BED | *X*^2^ (1, *N* = 47) = .33, p = .57 | Phi (Φ) = .13 | *FET* = .71 | Phi (Φ) = .13 |
|  | AN vs BN | *X*^2^ (1, *N* = 39) = 1.28, p = .26 | Phi (Φ) = .25 | *FET* = .42 | Phi (Φ) = .19 |
|  | BED vs BN | *X*^2^ (1, *N* = 32) = .08, p = .77 | Phi (Φ) = .12 | *X*^2^ (1, *N* = 29) = <.01, p = .1 | Phi (Φ) = .06 |
| Any Behavioural Disorder | AN vs no ED | *FET* = .05 | Phi (Φ) = .22 | *FET* = .003* | Phi (Φ) = .43 |
|  | BED vs no ED | *FET* = 1.00 | Phi (Φ) =.11 | *FET* = 1.00 | *-* |
|  | BN vs no ED | *FET* = .52 | Phi (Φ) = .06 | *FET* = .20 | Phi (Φ) = .26 |
|  | AN vs BED | *FET* = .06 | Phi (Φ) = .30 | *FET* = .10 | Phi (Φ) = .36 |
|  | AN vs BN | *FET* = .64 | Phi (Φ) = .13 | *FET* = .37 | Phi (Φ) = .20 |
|  | BED vs BN | *FET* = .38 | Phi (Φ) = .23 | *FET* = .41 | Phi (Φ) = .22 |
| Other Disorder | AN vs no ED | *FET* = .08 | Phi (Φ) = .21 | *FET* = .16 | Phi (Φ) = .20 |
|  | BED vs no ED | *FET* = .43 | Phi (Φ) = .09 | *FET* = .45 | Phi (Φ) = .10 |
|  | BN vs no ED | *FET* = 1.00 | Phi (Φ) = .05 | *FET* = 1.00 | Phi (Φ) = .06 |
|  | AN vs BED | *FET* = .62 | Phi (Φ) = .11 | *FET* = 1.00 | Phi (Φ) = .10 |
|  | AN vs BN | *FET* = .54 | Phi (Φ) = .19 | *FET* = .50 | Phi (Φ) = .23 |
|  | BED vs BN | *FET* = 1.00 | Phi (Φ) = .14 | *FET* = 1.00 | Phi (Φ) = .16 |

*AN: Anorexia Nervosa;*

*BED: Binge Eating Disorder;*

*BN: Bulimia Nervosa*;

*p: Significance level (.05);*

**: Significant after Bonferroni adjustment for multiple comparisons (p <= 0.05 / 6);*

*-: Could not compute*

S2. *Bonferroni-adjusted pairwise Wilcoxon Rank Sum* *Tests* *of diagnostic groups in terms of mental health problems between children at familial risk for eating disorders and healthy controls in the whole group*

S3*. Bonferroni-adjusted pairwise Wilcoxon Rank Sum* *Tests* *of diagnostic groups in terms of mental health problems between children at familial risk for eating disorders and healthy controls in the female group*

S4*. Age and group (FHR vs HC) as predictors of SDQ in children at familial risk for eating disorders: Odds Ratio (OR), Confidence Intervals (CI), p-value.*

|  | *Whole sample* | | *Female sample* | |
| --- | --- | --- | --- | --- |
|  | **Group** | **Age** | **Group** | **Age** |
| Emotional symptoms score | 4.62 [2.28-9.57],  p <.001* | 0.90 [0.78-1.05],  p = .18 | 5.93 [2.63-13.86],  p < .001 * | 0.95 [0.81-1.12],  p = .54 |
| Conduct problems score | 1.29 [0.65-2.59],  p = .46 | 0.97 [0.83-1.13],  p = .68 | 0.98 [0.44-2.18],  p = .96 | 0.96 [0.81-1.14],  p = .66 |
| Hyperactivity score | 1.99 [1.02-3.91],  p = .04* | 0.99 [0.86-1.16],  p =.96 | 1.85 [0.86-4.02],  p = .12 | 0.99 [0.84-1.17],  p = .89 |
| Peer problem score | 2.68 [1.33-5.48],  p = .006* | 0.98 [0.84-1,15],  p = .79 | 2.22 [0.97-4.69],  p = .06 | 0.98 [0.83-1.17]  p = .86 |
| Pro social score | 1.45 [0.73-2.90],  p = .29 | 1.00 [0.86-1.17],  p = .95 | 1.77 [0.81-3.91],  p = .15 | 1.05 [0.88-1,24]  p = .60 |
| Impact score | 5.77 [2.38-15.27],  p < .001* | 1.08 [0.89 – 1.31],  p = .44 | 5.45 [1.98-16.75],  p = .002* | 1.07 [0.86-1.33],  p = .55 |
| Total difficulties score | 3.52 [1.77-7.11],  p < .001* | 0.93 [0.80-1.09],  p = .34 | 3.56 [1.63-8.01],  p = .002* | 0.96 [0.81-1.13],  p = .61 |

*FHR: Familial High-Risk for eating disorders;*

*HC: Healthy Controls*;

**: p <= 0.05;*
